# Pathological and immunological characteristics of piglets infected experimentally with a HP-PRRSV TJ strain

**DOI:** 10.1186/s12917-016-0854-x

**Published:** 2016-10-12

**Authors:** Zhenguang Li, Yanliang He, Xiaoqin Xu, Xue Leng, Shufen Li, Yongjun Wen, Fengxue Wang, Mingqi Xia, Shipeng Cheng, Hua Wu

**Affiliations:** 1 Sinovet (Beijing) Biotechnology Co., Ltd., Kaituo Road 5, Haidian District, Beijing, 100085 China; 2 State Key Laboratory of Special Economic Animal Molecular Biology, Institute of Special Economic Animal and Plant Sciences, Chinese Academy of Agricultural Sciences,, Juye Street 4899, Changchun, Jilin 130122 China; 3 Jiangyan Animal Health Inspection Institute, Jiangguan Road 251, Taizhou, Jiangsu 225529 China

**Keywords:** HP-PRRSV, humoral immunity, cell-mediated immunity, immune responses

## Abstract

**Background:**

Porcine reproductive and respiratory syndrome (PRRS) remains a major threat to swine industry all over the world. The aim of this study was to investigate the mechanism of pathogenesis and immune responses caused by a highly pathogenic porcine reproductive and respiratory syndrome virus (HP-PRRSV).

**Results:**

All piglets experimentally infected with a HP-PRRSV TJ strain virus developed typical clinical signs of PRRS. The percentages of CD3^+^, CD4^+^, and CD8^+^ lymphocytes significantly decreased in the infected group as compared to the uninfected control animals (*p* < 0.01). Total WBC dropped in the infected animals during the experiment. The level of ELISA antibody against PRRSV increased in 7–10 days after infection and then started to decline. Pathological observations demonstrated various degree lesions, bleeding and necrosis in the lungs of the infected piglets.

**Conclusions:**

These results clearly indicated that HP-PRRSV TJ strain infection would activate host humoral immune response at the early period post infection and cause severe pathological damages on lungs and inhibit cellular immune response after infection.

## Background

Porcine reproductive and respiratory syndrome (PRRS) is characterized by reproductive failure in pregnant sows and respiratory distress in pigs of all ages [1]. The disease was first reported in the United States in 1987 and later in the Netherlands and other parts of the world [2]. The PRRS virus (PRRSV) is an enveloped positive-stranded RNA virus which belongs to the family of *Arteriviridae* in the order of the *Nidovirales*. Other related viruses of family *Arteriviridae* include equine arterutus virus, lactate dehydrogenase-elevating virus and simian haemorrhagic fever virus [3].

PRRSV mainly replicates in alveolar macrophages in the lungs and lymphoid organs [4]. Thus, the potential for aerosol spread through respiratory and oropharyngeal excretions within swine confinement units is a distinct possibility. The virus is well- known to stay in lungs and lymphoid organs of infected pigs for a long time. It was reported that an infected sow was able to transmit PRRSV up to 157 days post initial infection [5]. Others had detected PRRSV in lymph organs up to 132 days when the piglets were infected in the uterus [6]. PRRSV was also detected more than 180 days post-infection [7]. The mechanism of PRRSV persistence is not completely understood but is likely related to the emergence of viral variants which can escape host immune response [8].

PRRS has now emerged as the most prevalent disease of swine in the world. In the United States, annual loss due to PRRS is estimated at 560 million dollars [9]. In early 2006, a highly pathogenic disease emerged in some swine farms in Jiangxi province of China, and then spread rapidly to the rest of China [10]. This disease remains a major threat to swine industry all over the world [11]. Infected pigs of all ages presented with clinical signs including continuous high fever of above 41 °C, depression, dyspnea, anorexia, red discoloration of the ears and skin, conjunctivitis, mild diarrhea, shivering and limping. The morbidity rate was 50–100 % with mortality rate of 20–100 % [12]. Studies demonstrated that highly pathogenic porcine reproduction and respiratory syndrome virus (HP-PRRSV) was the major pathogen that caused the outbreak. HP-PRRSV TJ strain was originally isolated from a piglet that died of a “high fever” in Tianjin, China, in 2006, and it had the same characteristics as those of other HP-PRRSV strains observed in China. HP-PRRSV strain TJ was culturally passaged on MARC-145 cells for attenuation so that it could be used for the development of a modified live virus (MLV) vaccine [13]. Genetic analysis indicates that the HP-PRRSV isolated from China has a discontinuous deletion of 30 amino acids (AA) in non-structural protein 2 (Nsp2), compared with the North American type of PRRSV strain. However, the mechanisms contributing to the molecular pathogenesis of the HP-PRRSV have not been elucidated.

Some preliminary studies reported that PRRSV modulates the host immune responses and alters host gene expression [14–17]. In order to further investigate the immunological characteristics of HP-PRRSV, ten five-week-old pigs were experimentally infected with HP-PRRSV TJ strain and pathological changes, humoral and cell-mediated immune responses were evaluated in the present study.

## Results

### Clinical signs observations post infection

**Fig. 1:**
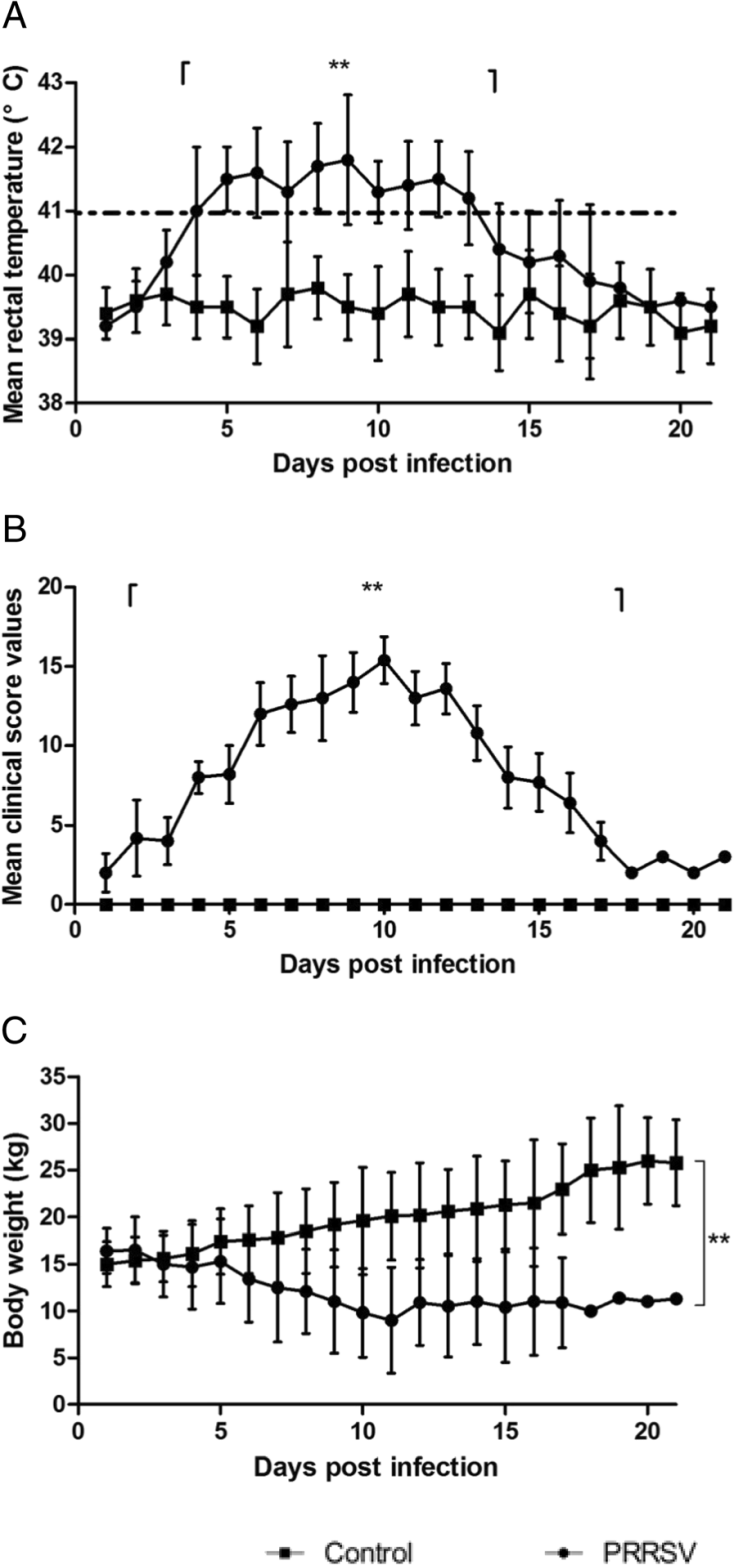
Clinical evaluation for each piglet post infection. After infection, mean rectal temperatures (**a**), mean clinical score (**b**) and body weight (**c**) of each animal were measured daily in HP-PRRSV inoculation group (PRRSV, *n* = 5) and control group (Control, *n* = 5). Rectal temperatures equal or above 41 °C (≥41 °C) were defined as fever. Data are presented as mean values ± SD. Asterisks indicate significant differences (**p < 0.01) between infected and control groups

All piglets infected with HP-PRRSV TJ strain virus developed typical clinical signs of HP-PRRS, such as severe depression and anorexia, lameness and shivering, dyspnea, skin cyanosis and death. Four of five PRRSV-infected piglets died of acute respiratory disease. Conversely, no clinical signs were observed in the control ones. Infected animals had persistently high fever (≥41 °C) at 4 day post infection (dpi), which lasted 9 days (Fig. 1a). In contrast, control piglets remained healthy with normal body temperature throughout the experiment. Animals in group 1 showed significantly higher average clinical scores than the control group (< 0.01) (Fig. 1b). As shown in Fig. 1c, animals infected with HP-PRRSV TJ strain in group 1 lost significantly more body weight than those in control group.

### Gross pathology and histological evaluations of lungs

**Fig. 2:**
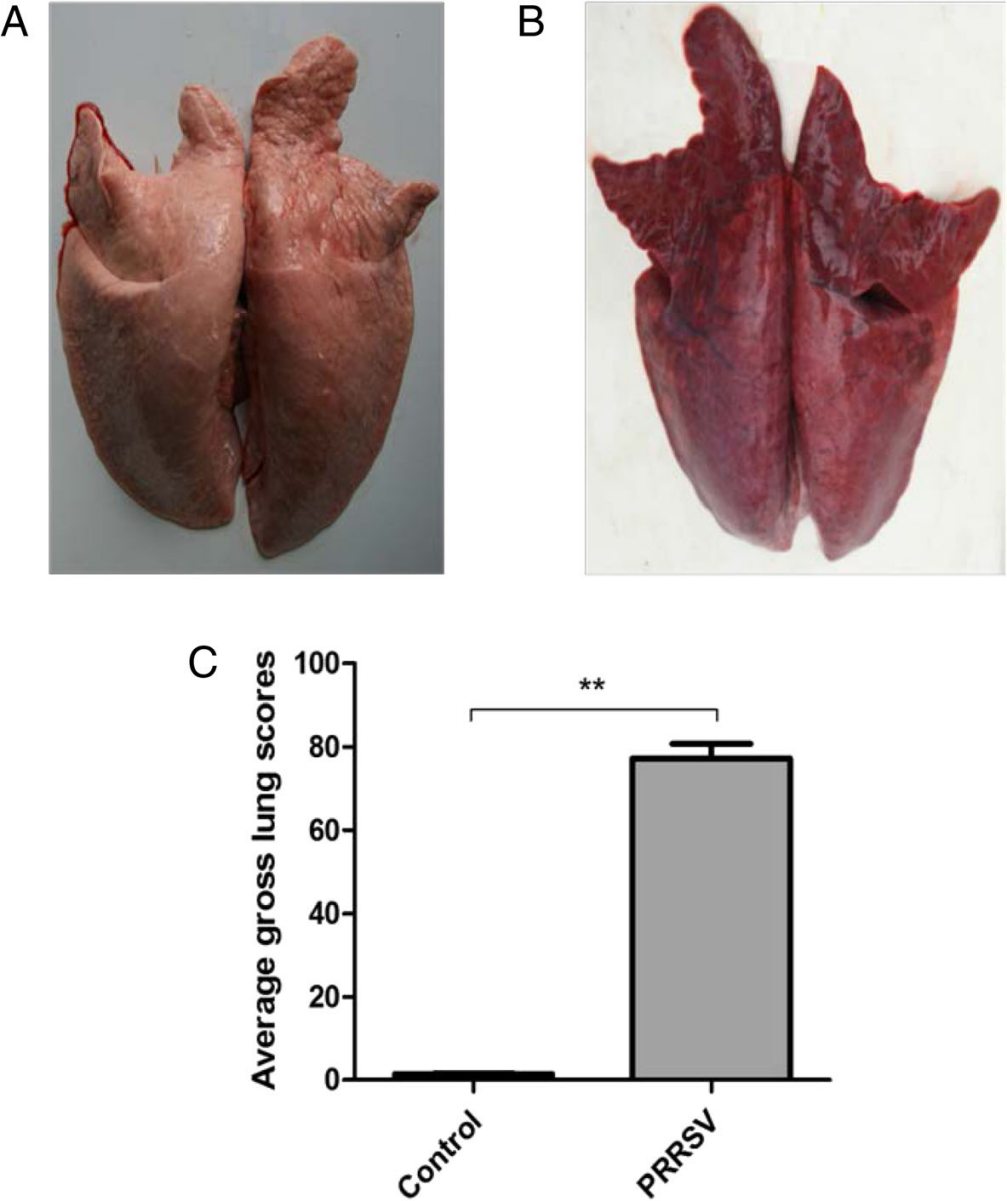
At necropsy, lungs of the control (**a**) and HP-PRRSV-infected (**b**) animals in the study were examined for macroscopic (gross) pathology. The average lung gross scores were evaluated according to the previous scoring system which estimates the percentage of lung affected by pneumonia in the two groups (*n* = 5). The difference of average lung gross scores between lungs of the control and HP-PRRSV-infected animals was presented (**c**). Data are presented as mean values ± SD. Asterisks indicate significant differences (**p < 0.01) between infected and control groups

No macroscopic (gross) lesions were recorded in the lungs collected from the control animals at necropsy (Fig. 2a). In HP-PRRSV-infected group, piglets exhibited severe gross lesions with consolidation and haemorrhage (Fig. 2b). The parenchymas of the infected lungs were firmer and heavier than those of the control group. The average lung lesion score was significantly higher (*p* < 0.01) than that in the control group (Fig. 2c).

**Fig. 3:**
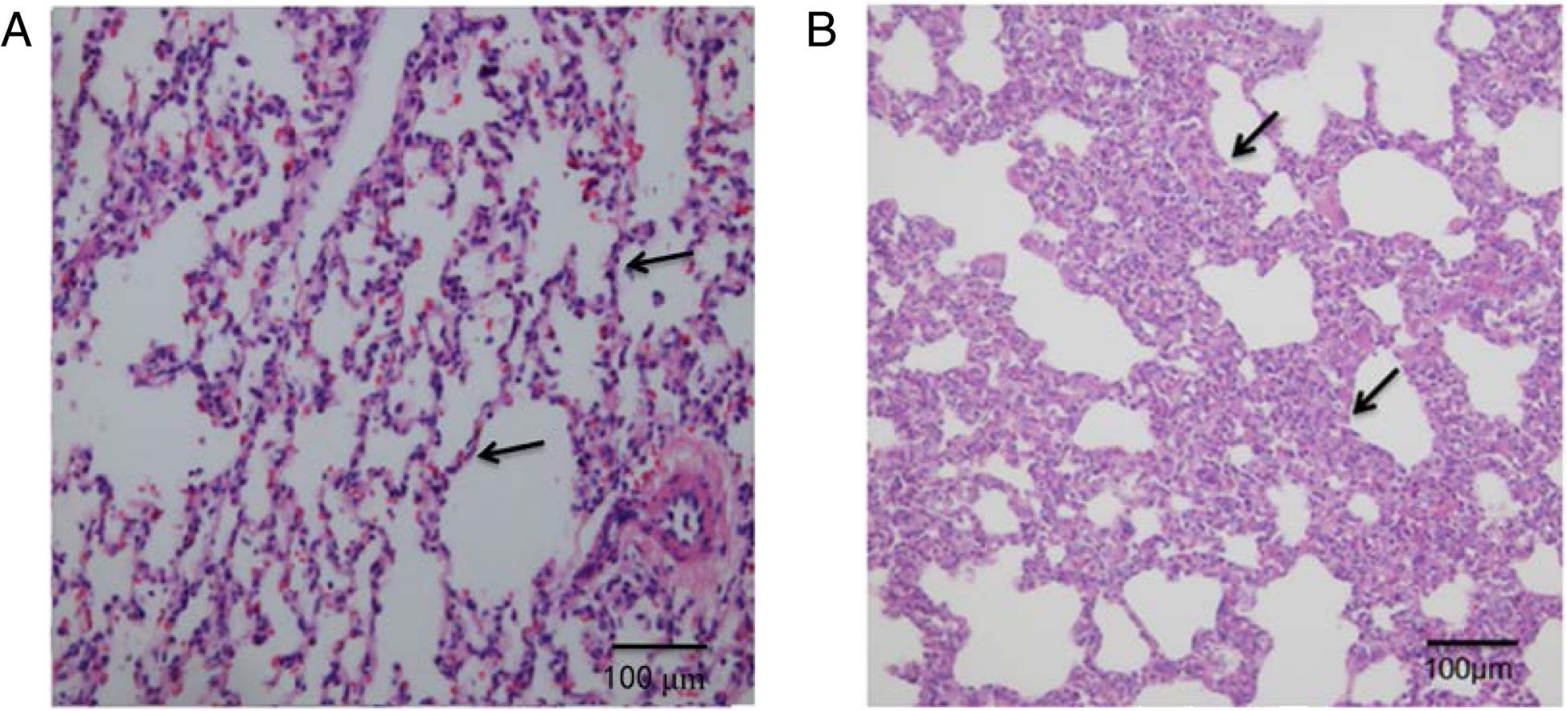
Histopathological lesions of the lung from HP-PRRSV infected and control piglets. At necropsy, lung samples were collected and processed for histopathology examination following hematoxylin and eosin (H&E) staining. Lungs of piglets infected with HP-PRRSV (**b**) showed severe interstitial pneumonia with thickened the alveolar septa (marked with arrows) accompanied by significant infiltration of mononuclear cells, compared to the lungs of negative control piglets (**a**)

Under histological examination, all the lungs from control piglets did not exhibit any features that are characteristic of acute PRRSV infection (Fig. 3a). However, the lung tissues collected from PRRSV-infected ones developed interstitial pneumonia with alveolar septa thickened by the infiltration of macrophages and lymphomononuclear cells (Fig. 3b).

### Leukocyte (white blood cell) count and antibody detection

**Fig. 4:**
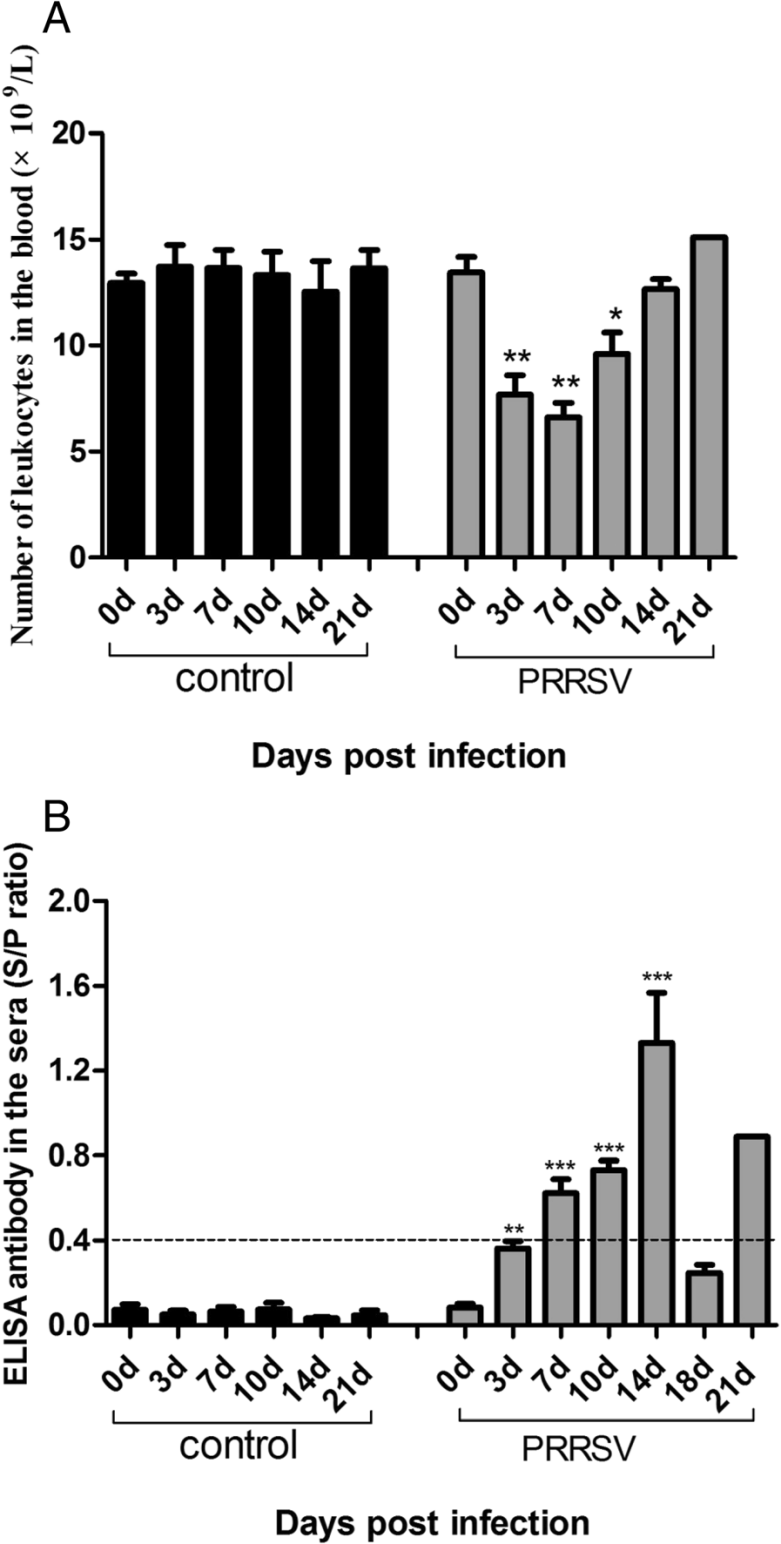
Leukocyte count and antibody detection in the sera from HP-PRRSV infected and control piglets. The heparinized blood sample from each piglet was collected for white blood cell counts (**a**); ELISA anti-PRRSV antibody titers in the sera were determined with the IDEXX PRRS ELISA kit (**b**). Values represent average titers from five animals. Dashed line at 0.4 S/P ratio designates threshold value above which titers are considered positive for anti-PRRSV antibodies. Data from groups of *n* = 5 piglets are expressed as means ± SD. The horizontal lines represent the geometric mean for each group and statistical analysis was performed and the data are shown as * *p* < 0.05, ** *p* < 0.01, *** *p* < 0.001

The average leukocyte levels during the study are shown in Fig. 4a. Piglets infected with HP-PRRSV TJ strain showed a sharp decrease in white blood cell counts (WBC) on 3 dpi, and reached the lowest level at 7 dpi. Then WBC developed a tendency to increase thereafter till 14 dpi. On 21 dpi, WBC level of the survival one returned to the normal level. In contrast, the WBC level in control group did not show significant change during the course of the experiment (Fig. 4a).

Antibody specific for PRRSV was first demonstrated by ELISA on 3 dpi in the infected group and increased gradually. However, ELISA antibody level showed a decline tendency after peak at 14 dpi. Low S/P ratio can be detected when animals died of infection, with the exception that the surviving one still showed a detectable titre when the experiment terminated. On the contrary, no detectable ELISA titre can be observed throughout the experiment in the control group (Fig. 4b).

### Alterations of peripheral T-lymphocyte subpopulations

**Fig. 5:**
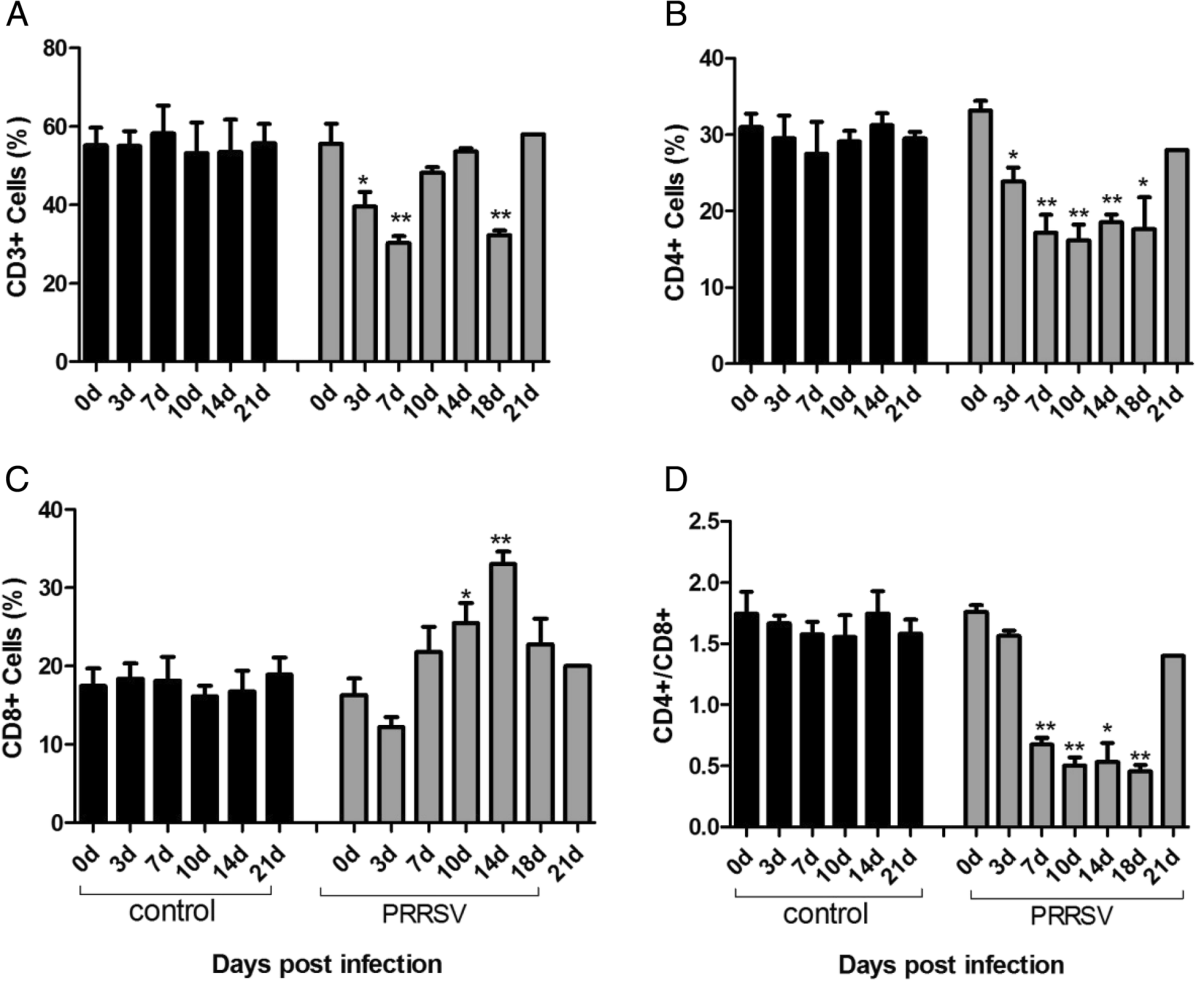
Percentage of T lymphocyte subsets in peripheral blood of HP-PRRSV infected and non-infected piglets. Piglets were infected with HP-PRRSV and blood was harvested after infection at 0, 3, 7, 10, 14, 18 and 21dpi. Single cell suspensions were prepared and stained with antibodies against T lymphocytes markers CD3, CD4, and CD8 and analyzed by flow cytometry. Representative flow cytometric plots of T lymphocytes are shown in the blood. The detailed analysis for CD3^+^ T lymphocytes are presented (**a**), CD4^+^ T lymphocytes (**b**), CD8^+^ T lymphocytes (**c**) and ratio of CD4^+^/CD8^+^ T lymphocytes (**d**). Data are shown as means ± SD. Asterisks indicate significant differences between the indicated experimental groups (*p < 0.05, **p < 0.01). Note: The CD4+ and CD8+ population are represented CD4+ CD8-cells and CD8+ CD4- respectively only

To investigate the influence of HP-PRRSV infection on peripheral blood T lymphocytes, T-lymphocyte subsets were evaluated using flow cytometry analysis. Before inoculation with the HP-PRRSV TJ strain, there was about 60 % CD3^+^ T- lymphocytes in the peripheral blood monouclear cells (PBMC) of piglets. As shown in Fig. 5a, the percentage of CD3^+^ cells significantly decreased at 3 (*p* < 0.05) and 7 dpi (*p* < 0.01), and returned to the similar level as before infection at 10 and 14dpi. However, the number of CD3^+^ cells showed dramatic decline when animals died of disease (*p* < 0.01). No significant change was detected for CD3^*+*^cells in the control group (Fig. 5a).

As shown in Fig. 5b, there was about 35 % CD4^+^ T lymphocyte in the PBMC of infected animals. Then the percentage sharply declined to about 22.7 % at 3 dpi and 15 % at 7 dpi. Interestingly, the proportion of CD4^+^ cells remained at a low level until 18 dpi in the PRRSV-infected group. Only the surviving one showed a normal level of CD4^+^ cells at 21 dpi. On the contrary, the control group remained unchanged throughout the experiment.

There was no significant difference of CD8^+^ cells in the control animals. As shown in Fig. 5c, there was about 19.4 % CD8^+^ T lymphocytes in PBMC prior to PRRSV infection. However, a transient decrease was observed at 3 dpi. Then there was a gradual increase in the percentage of CD8^+^ cells at 7–14 dpi. Finally, a slight decrease in the number of CD8^+^ T lymphocytes occured at 18 dpi, which is still higher than the normal level.

Figure 5d showed the CD4^+^/CD8^+^values in the both groups. The value kept about 1.5 during the study in the control animals. Similarly, in the infected group, the CD4^+^/CD8^+^value remained unchanged at 3 dpi. Nevertheless, the value significantly declined to about 0.6 at 7 dpi and peeked about 0.4 at 10 dpi and remained at a low level until 18 dpi in the PRRSV-infected group. Only the surviving one showed a normal CD4^+^/CD8^+^ value at 21 dpi.

## Discussion

PRRS has been a widespread disease in most pig-producing countries since its first emergence two decades ago in the USA [18]. Current studies have indicated that the emergence and epidemic of highly pathogenic PRRSV not only further aggravates the complicated situation of PRRS infection, but also enhances the genetic diversity of PRRSV in China [19]. Since its emergence in 2006, the HP-PRRS has been devastating in China’s swine industry. In current study, the newly isolated PRRSV TJ strain was used to experimentally infect piglets and reproduce the disease symptoms in which the clinical signs and mortality were virtually identical to what was seen in the field. The classical PRRS strains induce abortions in pregnant pigs, transient fever, depression and dyspnoea in male and young pigs [4]. Newborn piglets may die, but the mortality rate is generally low in growing pigs. It is intriguing that the new type of PRRSV that recently emerged in China exhibits unusually high mortality in affected pigs of all ages including adults. The pathogenesis of HP-PRRSV needs to be studied in detail in comparison to classical PRRS strains.

Previous studies demonstrated that HP-PRRSV infected pigs exhibited severe clinical symptoms including persistent high fever, red coloration of body, cyanopathy of ears, conjunctivitis, dyspnoea and severe diffuse pulmonary consolidation lesions [20, 21]. Pigs demonstrated significantly reduced numbers of pulmonary alveolar macrophages and peripheral blood monocytes, delayed neutralizing and non-neutralizing antibody response after PRRSV infection [22–26]. Piglets infected with HP-PRRSV TJ strain in our study developed the typical highly virulent PRRS clinical symptoms reported before. The number of white blood cell counts (WBC) sharply decreased at 3 dpi and peaked at 7 dpi post infection, even though it returned to increase thereafter.

During acute PRRSV infection, pigs developed significantly reduced humoral immune response to classical swine fever modified-live virus vaccine [27]. The immunosuppressive effects are most potent one week after PRRSV infection [28]. It is commonly speculated that HP-PRRSV strains and classical PRRSV strains have similarly destructive effects on the immune system. In present study, the fact that antibody specific for PRRSV was first demonstrated by ELISA at 3 dpi indicated that host humoral immune response was activated at the early period post infection. However, ELISA antibody level showed a dramatic decline after peak at 14 dpi. Undetectable S/P ratio was observed when animals died of disease, which indicated that host humoral immune response may be suppressed by PRRSV infection.

The present study reported the changes of subpopulations of T-lymphocytes in the HP-PRRSV challenged piglets. Pigs were reported to have transient lymphocytopenia of CD4^+^CD8^−^ and CD4^−^CD8^+^ T cells from three days to approximately four weeks after PRRSV infection [29–31]. The T-cell responses to PRRSV are induced 2–8 weeks post-infection [32, 33]. In our study, the percentages of CD3^+^ T lymphocytes subsets in the PBMCs and CD8^+^cells in the T lymphocytes both significantly decreased at 3 and/or 7 dpi, then had a tendency to increase until 14 dpi. Similarly, both of CD3^+^ and CD8^+^ cells declined to the low level at 18 dpi when animals died of disease. These results are consistent with Shimizu’s report that total lymphocytes, CD8^+^ cells decreased after PRRS virus infected [31]. Nevertheless, contradictory results showed that restimulation with PRRSV of PBMC derived from PRRSV infected pigs in vitro resulted in an increased percentage of CD3^+^CD8^high^ cells starting from 14 dpi compared to mock-restimulation. On the other hand, the number of CD4^+^ and CD4^+^/CD8^+^ value showed a similar tendency throughout the experiment period. They both decreased from 3 dpi to 7 dpi and remained the low level before animal died. Interestingly, all the T lymphocytes of the surviving one returned to normal level in the end of the study. These findings agreed with the Shimizu’s study. In contrast, previous study demonstrated that PRRSV increased the number of CD4^+^CD8^+^CD25^+^Foxp3^+^ cells at 14dpi, whereas CD4^+^CD8^−^CD25^+^Foxp3^+^ remained constant until 28 dpi [34]. In our study, CD4^+^CD8^+^ cells showed a transient increase from 1 to 3 dpi, though it returned to low level afterward (data not shown).

Results from this study suggest that HP-PRRSV TJ strain has more seriously destructive effects on the immune system than classical PRRSV strains, and induces seriously poor adaptive immune responses on the piglets, and facilitates the invasion of other pathogens.

## Conclusions

In this study, the experimental infection with HP-PRRSV TJ strain has reproduced the disease of high virulence and high mortality compared to the clinical signs seen in classical PRRS. It is indicated that HP-PRRSV TJ strain infection would activate host humoral immune response at the early period post infection and cause severe pathological damages on lungs and inhibit cellular immune response after infection.

However, comparison of pathogenesis between HP-PRRSV and LP-PRRSV infection need to be further investigated.

## Methods

### Cells, antibodies and viruses

The MARC-145 cell line (derived from African green monkey kidney cells) was employed for viral propagation and titration. MARC-145 cells were maintained in minimum essential medium (MEM) supplemented with 8 % fetal bovine serum (FBS, Hyclone Laboratories Inc, South Logan, UT, USA) at 37 °C, with 5 % CO2. PRRSV strain TJ (GenBank accession no. EU860248) was isolated and maintained in our laboratory as previously described [35]. Antibodies used for flow cytometric analysis, such as anti-CD3 (PE/Cy5-conjugated MAb PPT3), anti-CD4 (PE-conjugated MAb 74-12-4), and anti-CD8 (FITC-conjugated MAb 76-2-11) were purchased from BD Pharmingen (San Jose, CA).

### Animals and experimental design

Ten healthy crossbred piglets from a PRRSV-free herd were obtained, weaned, and transported to the biological safety level 2 (BSL2) facilities at the Teyan Veterinary Biologic Factory at three weeks of age. All animals were confirmed to be free of PRRSV, CSFV, PRV, SIV, *Mycoplasma hyopneumoniae*, and PCV2 infections using methods previously described (Leng et al. [13]). Weaned piglets were collectively housed for several days prior to exposure to virus, and then individually housed in the isolation units from the time of exposure until the end of the observation period. Ten piglets were randomly divided into two groups. Piglets of group 1 (*n* = 5) were inoculated intranasally (i.n.) with 10^3.0^ 50 % tissue culture infective doses (TCID_50_) of PRRSV TJ strain. Group 2 (*n* = 5) was inoculated intranasally with supernatant of lysate of MARC-145 cells in a 1-mL volume. All experimental procedures have been reviewed and approved by local Animal Care & Use Committee of Institute of Special Wild Economic Animal and Plant Sciences, Chinese Academy of Agricultural Sciences, Jilin, China.

### Clinical observation and sample collection

After infection, animals were monitored twice daily for clinical signs, including depression, anorexia, diarrhea, skin discolorations, conjunctivitis, lameness, coughing, labored and abdominal breathing and respiratory rate. Rectal temperatures were recorded every day throughout the experiment. Clinical signs and food intake were evaluated daily for each animal in both groups. Clinical signs were observed and graded using a scoring system described previously [36], which is shown in Table 1. Blood samples with and without heparin were collected on 0, 3, 7, 10 and 14 days post-infection (dpi). Heparinised blood samples were used to detect CD3^+^, CD4^+^, and CD8^+^ T lymphocytes and white blood cell counts. Serum samples were used to measure the ELISA antibody titers against PRRSV. All the animals were euthanized at day 21 dpi.

**Table 1:** Scores assigned to clinical signs observed in pigs following inoculation of HP-PRRSV

Category of clinical sign	Symptom	Score
Systemic:	Normal	0
	Apathy	1
	Anorexia	2
	Severe depression	5
	Death	10
Respiratory:	Normal	0
	Sneezing, Coughing	1
	Tachypnea	2
	Laboured breathing	3
	Abdominal breathing	5
Other symptoms:	Normal	0
	Diarrhea,	1
	Conjunctivitis	2
	Reddening of the ears	3
	Skin cyanopathy	5

### Serological examination

PRRSV-specific antibody responses were analyzed with serum collected at 0, 3, 7, 10, 14 and 21 dpi, using a commercial ELISA kit (Idexx Laboratories Inc., Westbrook, ME) according to the manufacturer’s instructions. The PRRSV-specific antibody level was reported as an S/P ratio, and the serum samples were considered positive if the S/P ratio was 0.4 or higher.

### Gross pathology and histological evaluations of lungs

At necropsy, lungs of the animals in the study were examined for macroscopic (gross) pathology, lungs were evaluated and graded using an established scoring system which estimates the percentage of lung affected by pneumonia. On the other hand, lung samples were collected and processed for histopathology examination following hematoxylin and eosin (H&E) staining, as described previously \^23^].

### Leukocyte (white blood cell) counts

Heparinised blood was collected and the leukocyte (white blood cell) counts were determined using an automatic analyzer CA 500 (Sysmex CA-500 automated blood coagulation analyzer, Japan) according to the manufacturer’s instructions.

### Flow cytometry analysis

Blood was collected and red blood cells were lysed with ACK lysis buffer (BioSource International, Inc., Camarillo, CA) for 1 min at room temperature. Single-cell suspensions (at 106 cells) were prepared in Hanks balanced salt solution (HBSS) (Invitrogen) and stained with antibodies against CD3, CD4, CD8 (BD PharMingen). After incubation on ice for 30 min, cells were washed twice in PBS containing 2 % FBS and 0.02 % NaN3. Then the cells were fixed with 1 % paraformaldehyde. Data collection and analysis were performed with a BD LSR-II flow cytometer, BD FACSDiva software (BD Pharmingen), FlowJo software (TreeStar, San Carlos, CA), Prism software, and Microsoft Excel (Seattle, WA).

### Statistical analyses

The significant differences of the in vitro experiment and animal trials were analyzed using a one-way or two-way ANOVA in the GraphPad Prism (version 5.0) software. A *t*-test or F-test was used to estimate the variability among the gross lesion, leukocyte count, antibody level and flow cytometry results. Differences were considered statistically significant at a P value of < 0.05 and extremely significant at a value of *P* < 0.01 or *P* < 0.001.

## Abbreviations

BSL2: biological safety level 2
dpi: days post-infection
FBS: fetal bovine serum
FCM: flow cytometry
H&E: Hematoxylin and eosin
HP-PRRSV: Highly pathogenic porcine reproductive and respiratory syndrome virus
MEM: Maintained in minimum essential medium
PBMC: Peripheral blood monouclear cells
PRRSV: Porcine reproductive and respiratory syndrome virus
TCID_50_: Tissue culture infective doses
WBC: White blood cells count
